# Epidemiological and genetic characteristics of porcine circovirus 3 in 15 provinces and municipalities of China between 2016 and 2020

**DOI:** 10.1186/s12985-022-01893-0

**Published:** 2022-11-14

**Authors:** Xugang Ku, Chengjun Zhang, Panpan Li, Xuexiang Yu, Qi Sun, Fengqin Xu, Ping Qian, Qigai He

**Affiliations:** 1 State Key Laboratory of Agricultural Microbiology, Huazhong Agricultural University, No.1, Shizishan Street, Hongshan District, Wuhan, 430070 Hubei Province People’s Republic of China; 2 The Cooperative Innovation Center for Sustainable Pig Production, Huazhong Agricultural University, No.1, Shizishan Street, Hongshan District, Wuhan, 430070 Hubei Province People’s Republic of China; 3 College of Veterinary Medicine, Huazhong Agricultural University, No.1, Shizishan Street, Hongshan District, Wuhan, 430070 Hubei Province People’s Republic of China

**Keywords:** Porcine circovirus 3, Phylogenetic analysis, Sequence alignment, PCR detection

## Abstract

Porcine circovirus 3 (PCV3) is a newly emerging virus and has been found associated with porcine dermatitis and nephropathy syndrome in pigs. Compared with PCV2, research into PCV3 cap gene sequencing is deficient. To investigate the prevalence and genotype distribution of PCV3, we collected 1291 samples from 211 pig farms throughout 15 provinces and municipalities. 312 out of 1291 samples were tested positive by PCR. We further sequenced and analyzed 164 PCR-positive samples. The majority (61.8%) of isolates we sequenced belong to genotype PCV3c. PCV3c is also the dominant genotype in Hubei, Hunan, Hebei province and Chongqing city. We found 3 sites under positive selection and located in predicted epitope peptide, revealing that the pig’s immunity may be a reason those sites are undergoing highly positive selection.

**Supplementary Information:**

The online version contains supplementary material available at 10.1186/s12985-022-01893-0.

## Introduction

Porcine circovirus 3 (PCV3) is the third circovirus identified in pigs which was first reported in 2016 in the United States using metagenomics [1].

PCV3 was first detected in pigs with cardiac and multi-systemic inflammation and was subsequently proven to have a relationship with Porcine Dermatitis and Nephropathy Syndrome (PDNS), as well as reproductive failure [2]. By challenged with infectious clone of PCV3, specific pathogen-free (SPF) pigs displayed PDNS-like clinical symptoms and subsequently suffered from lung, kidney, and lymph system damage [3]. Another experiment showed that PCV3-challenged mice presented symptoms of pneumonia [4]. PCV3 showed relatively high co-infection rates with porcine reproductive and respiratory syndrome virus (PRRSV), porcine parvovirus (PPV), and torque teno sus virus (TTSV) [5–7], which are associated with reproductive disorders.

Since its discovery, PCV3 has been detected in locations worldwide and has been found in samples tracing back to 1996 [8]. It has also been identified in wild boar, ticks, mosquitoes, and dogs [9–11]. These animals could act as reservoirs for PCV3, demonstrating this virus's complicated transmission circulation and origin. Therefore, PCV3 has already attracted a great deal of attention since it was discovered.

We previously conducted the first report in China on PCV3 [12], indicating the primary early infection information of PCV3 in the intensive pig farms, followed by whole genomic sequencing. The genome of PCV3 is circular, covalently closed, and consists of 2000 nucleotides. It has two major open reading frames: ORF1 encodes replication-associated proteins, while ORF2 encodes the Cap protein, which forms the capsid of the virus [2]. Compared with Porcine circovirus 2 (PCV2), research into PCV3 cap gene sequencing is deficient. To investigate the prevalence and genotype distribution of PCV3, we collected samples from 15 provinces and municipalities. We also sequenced 164 PCR-positive PCV3 samples and analyzed the epidemiological and genetic characteristics of those sequences.

## Material and method

### Sample collection and DNA extraction

1291 samples including hearts, spleens, kidneys, lungs, lymph nodes and anti-agglutinated blood were collected from 211 pig farms throughout 15 provinces and municipalities. Samples were resuspended in PBS (Phosphate-buffered saline) and homogenized by Qiagen TissueLyser II. Three Cycles of freezing and thawing were done to further rupture tissues and release viruses. After centrifuging under 13,400 × *g* for 10 min, 200 μL supernatant was used for DNA extraction by Tiangen.

### Polymerase chain reaction (PCR) detection and sequencing

A pair of primers was used to amplify the complete ORF2 gene of PCV3: PCV3-F: TTACTTAGAGAACGGACTTGTAACG, PCV3-R: AAATGAGACACAGAGCTATATTCAG. The reaction conditions were as follows: pre-denaturation at 94 °C for 5 min, 35 cycles of denaturation at 94 °C for 30 s, annealing at 55 °C for 30 s, extension at 72 °C for 1 min and a final extension at 72 °C for 10 min. PCV3-FS: ACATGCGAGGGCGTTTACCTGTG and PCV3-FR: CGGAGCATCCATAATGGGATACCAC were used to amplify an 840 bp amplicon(annealing at 57 °C, with the same reaction conditions as above PCR) contain the complete cap CDS gene and send to Sangon shanghai for Sanger sequencing. If more than one sample were positive from a farm tested positive, we would pick one sample for sequencing.

### Data analyses and prediction

Maxlikelyhood tree was constructed by IQTree2 [13]. Nucleotide and similarities were analyzed by MEGA [14]. Hyphy [15] was used to calculate selection pressure for each epitope site. Immune Epitope Database (IEDB) prediction tools [16] was used to predict potential epitope site.

## Results and discussion

In this study, 312 out of 1291 samples were tested positive, and we further collected and sequenced 164 PCR-positive samples which were collected 15 provinces and municipalities across China from 2016 to 2020. The positive rate is 24.17%, compared with existing researches, 12.2% of 616 samples in 21 Provinces of China during 2015–2017 [17]. 5% of 4040 tonsil samples which were collected from 89 farms in 25 provinces [18]. 86.7% of 491 Lung tissue samples from 19 pig slaughterhouses across 11 cities throughout Shanxi Province [19]. 63.14% of 36 large-scale pig farms were collected from 17 provinces [20]. 13.3% of 472 samples from domestic pigs were collected in Northeast China from 2015 to 2018 [21]. 28.4% of 2125 porcine samples from 910 cases in the Midwest of the USA were collected during 2016–2018 [22]. 36.70% of 79 tissue and serum samples from commercial farms in Brazil [6]. 20.5% of 49 tissue samples from Swedish pig herds. 44.2% of 360 samples from 73 pig farms in Korea [23]. Because of the sample collection location and sample type, the positive rate in different researches are very different. Our results only represent the positive rate of the sample we used.

We further analyze the positive rate in pigs at different age, and the results show that the positive rate is higher compared with the healthy pig in the nursery and grow stage,.reveal that pcv3 should be tested when the nursery and grow pig have emaciation, dermatitis or respiratory symptoms. In 222 aborted fetus samples we tested, 34.7% tested positive. A closely positive rate of Aborted fetuses also occurred in another research which was detected in 18/53 (33.9%) reproductive failure cases [24]. PCV3 should be considered and have further study when dealing with the abortion problem. The positive rate in healthy sows is 47%, higher than in healthy nursery and healthy grow pigs. More details are in Table 1.

**Table 1:** The PCV3 DNA positive rates in different age and healthy groups

Group	Positive sample	Totalsample number	Positive rate (%)
Nursery pigs with emaciation dermatitis or respiratory symptoms	28	176	15.9
Healthy Nursery pigs	10	183	5.4
Growing pigs with PDNS like symptom	32	198	16.2
Healthy Growing pigs	8	178	4.5
Aborted fetus	77	222	34.7
Healthy sows	157	334	47.0

**Fig. 1:**
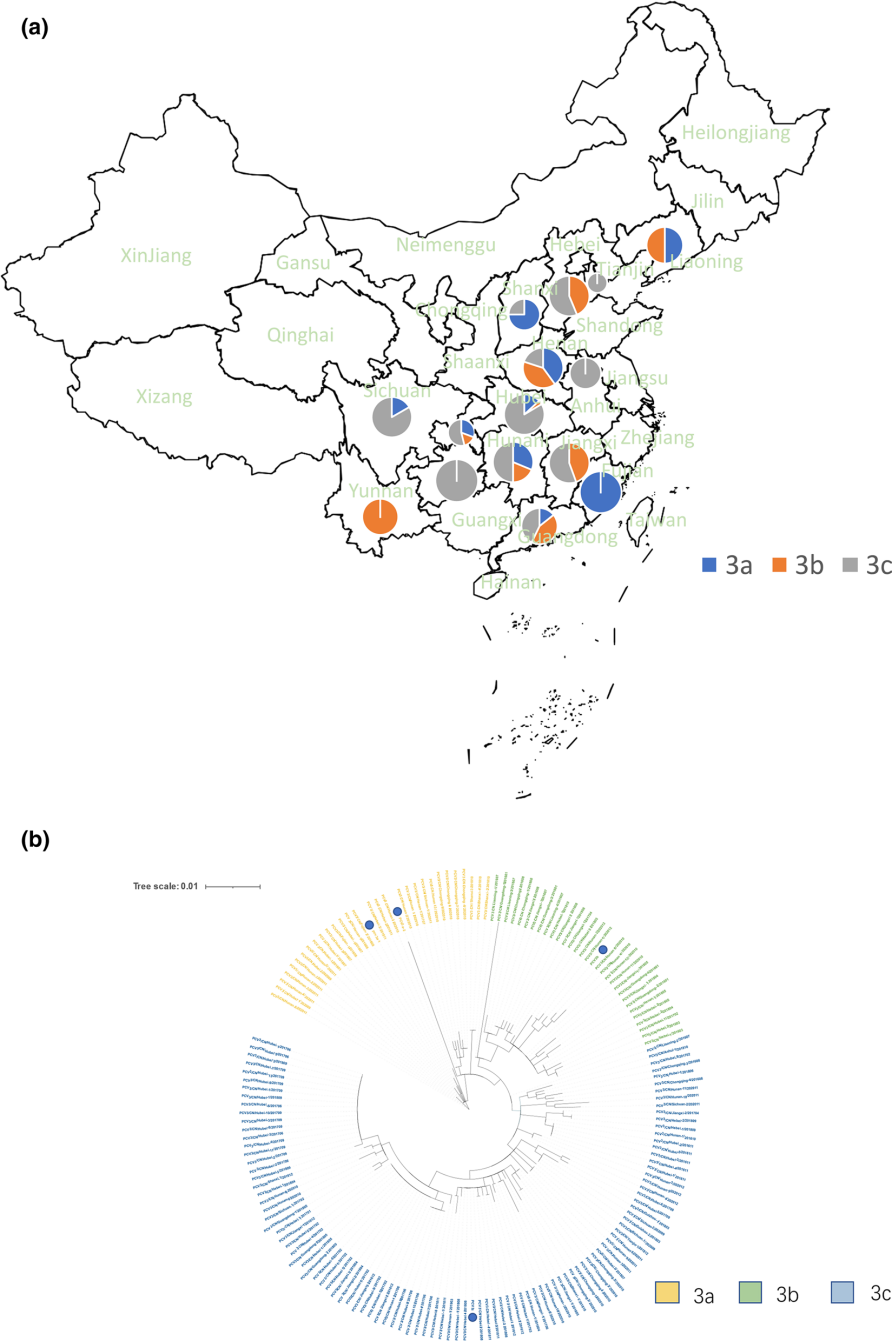
Geographical distribution and phylogenetic tree of different genotypes. **a** Distribution of different genotypes of PCV3 in different provinces. Provinces in which we have sequenced PCV3 isolates were marked as green. **b** Phylogenetic tree of our isolates with reference isolates of different genotypes. The reference strains was marked with blue dot

ORF2 sequence-based Maxlikelyhood tree was constructed by IQtree2, HKY + F + R2 were chosen as the models according to BIC, Testing tree branches by SH-like aLRT with 1000 replicates. Based on the phylogenetic analysis tree shown in Additional file 1: Fig. S1, all isolates can be divided into three branches [25], and the geographic distributions of the different genotypes are presented in Fig. 1. Results reveal the number of isolates belonging to subtype 3a, 3b and 3c are 33, 33, and 107 respectively. Thus, the majority (61.8%) of PCV3 isolates we sequenced belong to genotype PCV3c. Combined with geographical information, we could conclude that PCV3c is the dominant genotype in Hubei, Hunan, Hebei province and Chongqing city. Another research detected serum samples from 2,568 clinically healthy pigs from 36 large-scale pig farms were collected from 17 provinces (Hunan, Xinjiang, Jilin, Zhejiang, Jiangsu, Jiangxi, Guangxi, Hebei, Shandong, Shanxi, Anhui, Yunnan, Hubei, Inner Mongolia, Henan, Sichuan, and Guizhou) between 2019 and 2020 got the result of 86.96% (20/23) of PCV3 isolates were PCV3c strains[20].

Analysis of the nucleotide sequences of the ORF2 gene shows that the PCV3 strains we sequenced share 93.4–100% nucleotide similarities and 95–100% amino acid similarities. This indicates a relatively high mutation frequency in the PCV3 ORF2 gene.

Subsequently, 293 PCV3 ORF2 gene sequences were downloaded from the National Center for Biotechnology Information (NCBI) database. According to the results shown in Additional file 1: Table S2, sites 5, 24, and 137 were proved under positive selection by the four methods of single likelihood ancestor counting, mixed effects model of evolution, fast unconstrained Bayesian approximation, and fixed effects likelihood. Sites 3, 156, and 214 were shown to be positively selected by two methods. The sites that tested positive using more than three methods can be considered as robust positive selection sites. Site 24 was also detected under positive selection by the other researchers [26, 27]. Positive selection corresponds with increasing virus fitness and is one of the main evolution forces [28, 29]. The designs of PCR detection primers and epitope vaccines should consider the positive select effect of these specific sites because these sites may have associated with virus fitness and higher mutation rate.

Using the Immune Epitope Database (IEDB) prediction tools, we found seven potential peptides more likely to be epitopes (Additional file 1: Table S3). Combined with the positive selection information we obtained in the previous section, we determined that site 5 and site 24, which are positive selection sites, are also found in the second prediction epitope peptide, while site 137 is the fifth peptide. These results reveal that the immunity antibody may be the reason why those sites are undergoing highly positive selection, considering the high positive rates (56.6% in the USA, 52.6% in Zhejiang province) of the PCV3 antibody in serum in some publications[30, 31]. The sites under the high positive selective pressure may correspond with increasing virus fitness of hosts to make PCV3 more adaptive to the host immunity system. The biological significance and the role of the positively selected site should be paid more attention to in further studies. Considering that some clinical cases and animal infection experiments have confirmed that PDNS and the injury of lymph, which is the crucial immune organ, are significant damage, pcv3 may evolve with more damage when its more adaptive to the pig host immunity system.

So far, there is no commercial vaccine against PCV3. Since PCV3 has a relatively high positive rate and is under immune selective pressure, PCV3 may increase its immune evasion capacity through mutation. Therefore, vaccine and anti-viral drug development should be put on the agenda, and the site under positive selection should be considered when developing a vaccine.

Compared with PCV2, research into PCV3 cap gene sequencing is deficient. Our sequencing data enriches the PCV3 ORF2 data, and all the sequencing data have been uploaded to the NCBI GenBank database and can be used by other researchers for further analysis. However, a more detailed molecular characterization of PCV3 is required to monitor and study its evolution pattern to develop more specific vaccines like subunits, peptides, DNA, and RNA vaccines.

In this study, we tested and sequenced samples from 15 regions across China to gather the necessary data that can be used to indicate PCV3 epidemiology in China. This can help us to understand the worldwide distribution of this virus and provide information helping the development of sensitive and specific diagnostic tools and vaccines.

## Biosecurity statement

All the virus detection procedure was performed in BSL-2 laboratory. The rest tissue sample and amplified product was heat-inactivated before disposal.

## Supplementary Information

**Additional file 1: Table S1.** Isolate geographic information and GeneBank accession number. **Table S2.** The result of positive/diversifying selection analyze results by different method. **Table S3.** Potential B cell peptides predicted by IEDB tools. **Fig. S1.** Nucleotide sequence consistency pattern of our isolates.

## Data Availability

The gene sequences sequenced during the current study are available in the GenBank database (accession numbers were in supplementary materials).
