# Supplementary material for: Epidemiological and genetic characteristics of porcine circovirus 3 in 15 provinces and municipalities of China between 2016 and 2020
Source: Virol J. 2022 Nov 14;19:187. doi: 10.1186/s12985-022-01893-0 (PMC9661739; doi:10.1186/s12985-022-01893-0)
Supplement: Supplementary file 1 — Additional file 1: Table S1. Isolate geographic information and GeneBank accession number. Table S2. The result of positive/diversifying selection analyze results by different method. Table S3. Potential B cell peptides predicted by IEDB tools. Fig. S1. Nucleotide sequence consistency pattern of our isolates. [file 12985_2022_1893_MOESM1_ESM.docx]

Table S1: Isolate geographic information and GeneBank accession number

| Isolates | Provinces | Genbank accession number |
| --- | --- | --- |
| PCV3/CN/Anhui-1/201810 | Anhui | MW089611 |
| PCV3/CN/Chongqing-1/201608 | Chongqing | MW089507 |
| PCV3/CN/Chongqing-1/202010 | Chongqing | OL619345 |
| PCV3/CN/Chongqing-2/201608 | Chongqing | MW089508 |
| PCV3/CN/Chongqing-2/202010 | Chongqing | OL619335 |
| PCV3/CN/Chongqing-3/201608 | Chongqing | MW089509 |
| PCV3/CN/Chongqing-3/202010 | Chongqing | OL619336 |
| PCV3/CN/Chongqing-4/201608 | Chongqing | MW089510 |
| PCV3/CN/Chongqing-4/202010 | Chongqing | OL619337 |
| PCV3/CN/Chongqing-5/202010 | Chongqing | OL619338 |
| PCV3/CN/Chongqing-6/202010 | Chongqing | OL619342 |
| PCV3/CN/Chongqing-7/202010 | Chongqing | OL619343 |
| PCV3/CN/Chongqing-8/202010 | Chongqing | OL619344 |
| PCV3/CN/Chongqing-9/202010 | Chongqing | OL619346 |
| PCV3/CN/Fujian-1/201608 | Fujian | MW089500 |
| PCV3/CN/Fujian-2/201608 | Fujian | MW089501 |
| PCV3/CN/Guangdong-1/201601 | Guangdong | MW089519 |
| PCV3/CN/Guangdong-1/201805 | Guangdong | MW089582 |
| PCV3/CN/Guangdong-2/201601 | Guangdong | MW089520 |
| PCV3/CN/Guangdong-2/201805 | Guangdong | MW089583 |
| PCV3/CN/Guangdong-3/201601 | Guangdong | MW089521 |
| PCV3/CN/Guangdong-3/201805 | Guangdong | MW089584 |
| PCV3/CN/Guangdong-4/201601 | Guangdong | MW089522 |
| PCV3/CN/Guizhou-1/201803 | Guizhou | MW089575 |
| PCV3/CN/Guizhou-2/201803 | Guizhou | MW089576 |
| PCV3/CN/Hebei-1/201603 | Hebei | MW089516 |
| PCV3/CN/Hebei-1/201612 | Hebei | MW089528 |
| PCV3/CN/Hebei-1/201701 | Hebei | MW089530 |
| PCV3/CN/Hebei-1/201703 | Hebei | MW089536 |
| PCV3/CN/Hebei-1/201804 | Hebei | MW089577 |
| PCV3/CN/Hebei-1/201805 | Hebei | MW089586 |
| PCV3/CN/Hebei-1/201807 | Hebei | MW089591 |
| PCV3/CN/Hebei-1/201808 | Hebei | MW089595 |
| PCV3/CN/Hebei-1/201809 | Hebei | MW089601 |
| PCV3/CN/Hebei-1/201810 | Hebei | MW089610 |
| PCV3/CN/Hebei-1/201811 | Hebei | MW089612 |
| PCV3/CN/Hebei-2/201603 | Hebei | MW089517 |
| PCV3/CN/Hebei-2/201612 | Hebei | MW089531 |
| PCV3/CN/Hebei-2/201804 | Hebei | MW089578 |
| PCV3/CN/Hebei-2/201805 | Hebei | MW089587 |
| PCV3/CN/Hebei-2/201808 | Hebei | MW089596 |
| PCV3/CN/Hebei-2/201809 | Hebei | MW089602 |
| PCV3/CN/Hebei-2/201811 | Hebei | MW089613 |
| PCV3/CN/Hebei-3/201805 | Hebei | MW089589 |
| PCV3/CN/Hebei-3/201808 | Hebei | MW089599 |
| PCV3/CN/Hebei-3/201811 | Hebei | MW089614 |
| PCV3/CN/Hebei-4/201808 | Hebei | MW089600 |
| PCV3/CN/Hebei-4/201811 | Hebei | MW089615 |
| PCV3/CN/Hebei-5/201811 | Hebei | MW089616 |
| PCV3/CN/Hebei-6/201811 | Hebei | MW089617 |
| PCV3/CN/Henan-1/201608 | Henan | MW089502 |
| PCV3/CN/Henan-1/201706 | Henan | MW089552 |
| PCV3/CN/Henan-1/201707 | Henan | MW089551 |
| PCV3/CN/Henan-1/201805 | Henan | MW089588 |
| PCV3/CN/Henan-1/201903 | Henan | MW089619 |
| PCV3/CN/Hubei-1/201601 | Hubei | MW089518 |
| PCV3/CN/Hubei-1/201611 | Hubei | MW089523 |
| PCV3/CN/Hubei-1/201702 | Hubei | MW089541 |
| PCV3/CN/Hubei-1/201706 | Hubei | MW089553 |
| PCV3/CN/Hubei-1/201709 | Hubei | MW089563 |
| PCV3/CN/Hubei-1/201806 | Hubei | MW089590 |
| PCV3/CN/Hubei-1/201807 | Hubei | MW089592 |
| PCV3/CN/Hubei-1/201809 | Hubei | MW089603 |
| PCV3/CN/Hubei-1/202009 | Hubei | OL619331 |
| PCV3/CN/Hubei-10/201706 | Hubei | MW089562 |
| PCV3/CN/Hubei-10/201709 | Hubei | MW089572 |
| PCV3/CN/Hubei-11/201702 | Hubei | MW089550 |
| PCV3/CN/Hubei-11/201709 | Hubei | MW089573 |
| PCV3/CN/Hubei-13/201709 | Hubei | MW089574 |
| PCV3/CN/Hubei-2/201611 | Hubei | MW089524 |
| PCV3/CN/Hubei-2/201702 | Hubei | MW089542 |
| PCV3/CN/Hubei-2/201706 | Hubei | MW089554 |
| PCV3/CN/Hubei-2/201709 | Hubei | MW089564 |
| PCV3/CN/Hubei-2/201807 | Hubei | MW089593 |
| PCV3/CN/Hubei-2/201809 | Hubei | MW089604 |
| PCV3/CN/Hubei-2/202009 | Hubei | OL619332 |
| PCV3/CN/Hubei-3/201611 | Hubei | MW089525 |
| PCV3/CN/Hubei-3/201702 | Hubei | MW089543 |
| PCV3/CN/Hubei-3/201706 | Hubei | MW089555 |
| PCV3/CN/Hubei-3/201709 | Hubei | MW089565 |
| PCV3/CN/Hubei-3/201807 | Hubei | MW089594 |
| PCV3/CN/Hubei-3/201809 | Hubei | MW089605 |
| PCV3/CN/Hubei-4/201611 | Hubei | MW089526 |
| PCV3/CN/Hubei-4/201702 | Hubei | MW089544 |
| PCV3/CN/Hubei-4/201706 | Hubei | MW089556 |
| PCV3/CN/Hubei-4/201709 | Hubei | MW089566 |
| PCV3/CN/Hubei-5/201611 | Hubei | MW089527 |
| PCV3/CN/Hubei-5/201702 | Hubei | MW089545 |
| PCV3/CN/Hubei-5/201706 | Hubei | MW089557 |
| PCV3/CN/Hubei-5/201709 | Hubei | MW089567 |
| PCV3/CN/Hubei-6/201702 | Hubei | MW089546 |
| PCV3/CN/Hubei-6/201706 | Hubei | MW089558 |
| PCV3/CN/Hubei-6/201709 | Hubei | MW089568 |
| PCV3/CN/Hubei-7/201702 | Hubei | MW089547 |
| PCV3/CN/Hubei-7/201706 | Hubei | MW089559 |
| PCV3/CN/Hubei-7/201709 | Hubei | MW089569 |
| PCV3/CN/Hubei-8/201702 | Hubei | MW089548 |
| PCV3/CN/Hubei-8/201706 | Hubei | MW089560 |
| PCV3/CN/Hubei-8/201709 | Hubei | MW089570 |
| PCV3/CN/Hubei-9/201702 | Hubei | MW089549 |
| PCV3/CN/Hubei-9/201706 | Hubei | MW089561 |
| PCV3/CN/Hubei-9/201709 | Hubei | MW089571 |
| PCV3/CN/Hunan-1/201604 | Hunan | MW089515 |
| PCV3/CN/Hunan-1/201610 | Hunan | MW089511 |
| PCV3/CN/Hunan-1/202010 | Hunan | OL619339 |
| PCV3/CN/Hunan-1/202011 | Hunan | OL619357 |
| PCV3/CN/Hunan-1/202012 | Hunan | OL619370 |
| PCV3/CN/Hunan-10/202010 | Hunan | OL619353 |
| PCV3/CN/Hunan-10/202011 | Hunan | OL619368 |
| PCV3/CN/Hunan-11/202010 | Hunan | OL619354 |
| PCV3/CN/Hunan-11/202011 | Hunan | OL619369 |
| PCV3/CN/Hunan-12/202010 | Hunan | OL619355 |
| PCV3/CN/Hunan-13/202010 | Hunan | OL619356 |
| PCV3/CN/Hunan-2/202010 | Hunan | OL619340 |
| PCV3/CN/Hunan-2/202011 | Hunan | OL619358 |
| PCV3/CN/Hunan-2/202012 | Hunan | OL619371 |
| PCV3/CN/Hunan-3/202010 | Hunan | OL619341 |
| PCV3/CN/Hunan-3/202011 | Hunan | OL619359 |
| PCV3/CN/Hunan-3/202012 | Hunan | OL619372 |
| PCV3/CN/Hunan-4/202010 | Hunan | OL619347 |
| PCV3/CN/Hunan-4/202011 | Hunan | OL619360 |
| PCV3/CN/Hunan-4/202012 | Hunan | OL619373 |
| PCV3/CN/Hunan-5/202010 | Hunan | OL619348 |
| PCV3/CN/Hunan-5/202011 | Hunan | OL619361 |
| PCV3/CN/Hunan-5/202012 | Hunan | OL619374 |
| PCV3/CN/Hunan-6/202010 | Hunan | OL619349 |
| PCV3/CN/Hunan-6/202011 | Hunan | OL619362 |
| PCV3/CN/Hunan-7/202010 | Hunan | OL619350 |
| PCV3/CN/Hunan-7/202011 | Hunan | OL619363 |
| PCV3/CN/Hunan-8/202010 | Hunan | OL619351 |
| PCV3/CN/Hunan-8/202011 | Hunan | OL619364 |
| PCV3/CN/Hunan-9/202010 | Hunan | OL619352 |
| PCV3/CN/Hunan-9/202011 | Hunan | OL619365 |
| PCV3/CN/Jiangxi-1/201605 | Jiangxi | MW089514 |
| PCV3/CN/Jiangxi-1/201607 | Jiangxi | MW089505 |
| PCV3/CN/Jiangxi-1/201612 | Jiangxi | MW089532 |
| PCV3/CN/Jiangxi-1/201704 | Jiangxi | MW089537 |
| PCV3/CN/Jiangxi-1/201706 | Jiangxi | MW089540 |
| PCV3/CN/Jiangxi-1/201804 | Jiangxi | MW089579 |
| PCV3/CN/Jiangxi-1/201805 | Jiangxi | MW089585 |
| PCV3/CN/Jiangxi-1/201808 | Jiangxi | MW089597 |
| PCV3/CN/Jiangxi-2/201608 | Jiangxi | MW089506 |
| PCV3/CN/Jiangxi-2/201612 | Jiangxi | MW089533 |
| PCV3/CN/Jiangxi-2/201704 | Jiangxi | MW089538 |
| PCV3/CN/Jiangxi-2/201804 | Jiangxi | MW089580 |
| PCV3/CN/Jiangxi-2/201808 | Jiangxi | MW089598 |
| PCV3/CN/Jiangxi-3/201612 | Jiangxi | MW089535 |
| PCV3/CN/Jiangxi-3/201704 | Jiangxi | MW089539 |
| PCV3/CN/Jiangxi-3/201804 | Jiangxi | MW089581 |
| PCV3/CN/Liaoning-1/201607 | Liaoning | MW089503 |
| PCV3/CN/Liaoning-2/201607 | Liaoning | MW089504 |
| PCV3/CN/Liaoning-3/201607 | Liaoning | MW089512 |
| PCV3/CN/Liaoning-4/201607 | Liaoning | MW089513 |
| PCV3/CN/Shanxi-1/201612 | Shanxi | MW089529 |
| PCV3/CN/Shanxi-1/201810 | Shanxi | MW089606 |
| PCV3/CN/Shanxi-2/201810 | Shanxi | MW089607 |
| PCV3/CN/Shanxi-3/201810 | Shanxi | MW089608 |
| PCV3/CN/Shanxi-4/201810 | Shanxi | MW089609 |
| PCV3/CN/Sichuan-1/201702 | Sichuan | MW089534 |
| PCV3/CN/Sichuan-1/202009 | Sichuan | OL619333 |
| PCV3/CN/Sichuan-1/202011 | Sichuan | OL619366 |
| PCV3/CN/Sichuan-2/202009 | Sichuan | OL619334 |
| PCV3/CN/Sichuan-2/202011 | Sichuan | OL619367 |
| PCV3/CN/Tianjin-1/201812 | TianJIn | MW089618 |

Table S2: The result of positive/diversifying selection analyze results by different method

| Method | positive/diversifying Amin acid selection site |
| --- | --- |
| Single-Likelihood Ancestor Counting | 137  5  24 |
| Mixed Effects Model of Evolution | 3  214  137  5  156  24  20 |
| Fast Unconstrained Bayesian AppRoximation | 137  5  156  214  150  3  24  56 |
| Fixed Effects Likelihood | 137  5  24 |

Table S3: Potential B cell peptides predicted by IEDB tools.

| Start | End | Peptide | Length |
| --- | --- | --- | --- |
| 5 | 25 | AIFRRRPRPRRRRRHRRRYAR | 21 |
| 42 | 61 | KYSTMNVISVGTPQNNKPWH | 20 |
| 71 | 81 | EWETAISFEYY | 11 |
| 94 | 105 | ISPAQQTKTMFG | 12 |
| 113 | 159 | GAWTTNTWLQDDPYAESSTRKVMTSKKKHSRYFTPKPILAGTTTAHP | 47 |
| 169 | 178 | PTPWLNTYDP | 10 |
| 189 | 204 | IYVPEKTGMTDFYGTK | 16 |


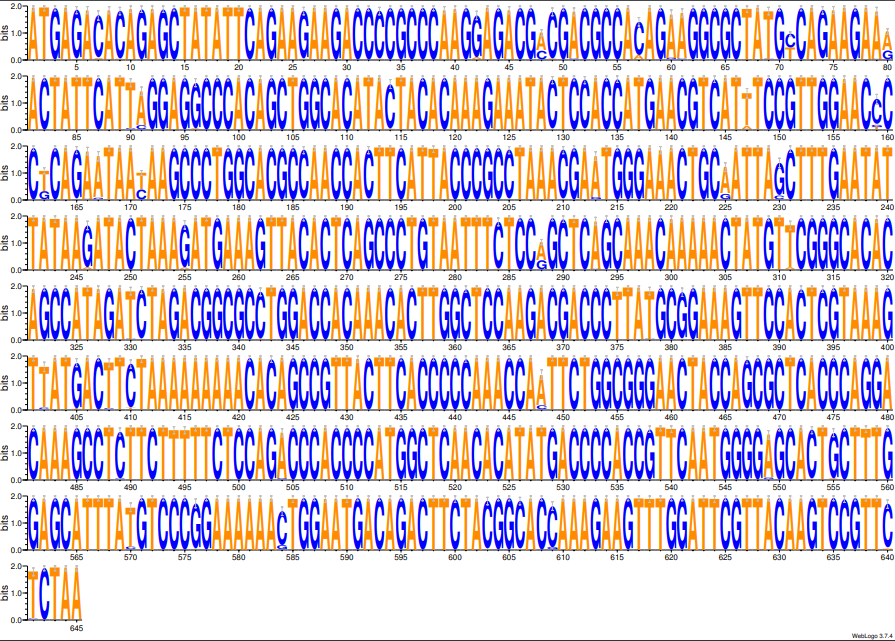


FigS1 Nucleotide sequence consistency pattern of our isolates.
